# CT features of retroperitoneal solitary fibrous tumor: report of three cases and review of the literature

**DOI:** 10.1186/1477-7819-12-324

**Published:** 2014-10-28

**Authors:** Jun Sun, Xiang-rong Yu, Bin-bin Shi, Jin Zheng, Jing-tao Wu

**Affiliations:** Department of Radiology, Northern Jiangsu People’s Hospital, Yangzhou University, 98, Nantong West Road, Yangzhou, 225001 China; Department of Radiology, Huashan Hospital, Fudan University, 12, Wulumuqi Middle Road, Shanghai, 200041 China

**Keywords:** Retroperitoneum, Solitary fibrous tumors, Tomography, X-ray computed

## Abstract

CT findings in three cases with solitary fibrous tumors (SFTs) confirmed by histopathology and immunohistochemistry were reviewed retrospectively, and compared with pathological results. The three tumors were large, well-defined, and smooth contour masses and SFT consisted of solid components of two different densities. On enhanced CT scans, tumors were strongly enhancing, the multiple vascular shadows were seen within the tumor in the arterial phase. There is progressive enhancement from the arterial to the venous phase, and the tumor capsule can be observed. Histologically, the tumors are composed of spindle cells within a background of collagen stroma, and showed a wide range of growth patterns, alternating hypercellular (tumor cell-rich) and hypocellular (collagen-rich) areas. The diagnosis is confirmed by characteristic positive immunohistochemical staining for CD34.

## Background

Solitary fibrous tumors (SFTs) are rare spindle-shaped cell tumors originated from dendritic mesenchymal cells. They mostly occur in visceral pleura, but rarely in the retroperitoneum [1, 2]. In the retrospective analysis of three cases of pathologically confirmed retroperitoneal SFTs in this article, their CT imaging and pathology were studied for the purpose of further improving the diagnostic accuracy in retroperitoneal SFTs.

## Case presentations

### Case one

**Figure 1:**
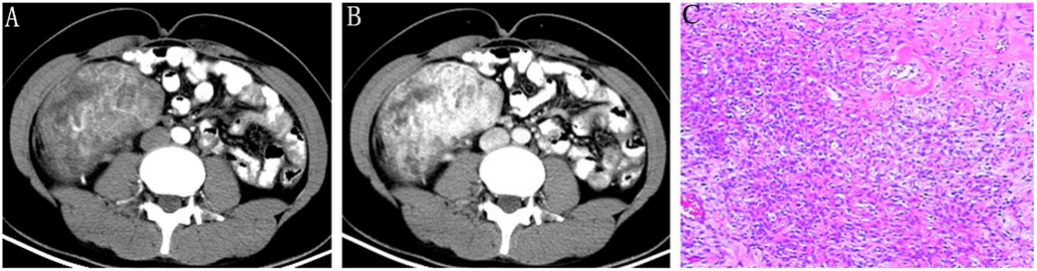
**Retroperitoneal solitary fibrous tumor in a 22-year-old woman. (A)** Solid part of the tumor in arterial phase is in moderate, uneven enhancement with extensive vessel, and unenhanced low-density cystic areas are visible inside. **(B)** The solid part of the tumor in the venous phase is insignificant, with uneven or no enhancement in low-density cystic areas, but envelope enhancement is visible. **(C)** Tumor cells with admixed thin and thick, collagen fibers. There is a relative demarcation between the hypercellular and hypocellular areas under the microscope.

Case one was a 22-year-old female who suffered from intermittent abdominal pain in central right position for 6 years, with paroxysmal pain radiating to the right waist, which relieved after resting. Upon physical examination, a palpable tough-quality mass in central right abdomen was seen, with no tenderness and rebound tenderness. Enhanced abdominal CT showed a lobulated soft tissue mass on the right retroperitoneum, 160 mm × 105 mm × 66 mm in size and with a clear boundary. The solid part of the tumor in the arterial phase showed a moderately heterogeneous enhancement, with a CT value of about 76 HU. A plexiform disordered vascular shadow was visible, together with extensive unenhanced low-density cystic areas (Figure 1A). A sustained and heterogeneously enhanced solid part of the scanned mass was observed in both the venous and delayed phase, with a CT value of about 152 to 181 HU. There was no enhancement for low-density cystic areas, although envelope enhancement was seen, with a CT value of about 27 to 30 HU (Figure 1B). The surrounding structures shifted under compression. Surgical resection was performed using a retroperitoneal approach, and a lobulated mass on the right retroperitoneum was visible in surgery. It enveloped the right ureter, showing a clear boundary with the kidney but no significant adhesions. With regards to surgical pathology, the gross morphology showed an envelope on the tumor surface, with a texturized gray cut surface and a fish-like pattern in the local area. Two regions within the tumor were visible under the microscope, with the focus area composed of spindle cells, which were dense and in a bundle, weaving arrangement. There were sparse cells in the focus area, with obvious edema (Figure 1C). Immunohistochemistry showed CD34, CD99 (+++), SMA, CK, NSE, Calretinin (-), S-100, and bcl-2 (+).

### Case two

**Figure 2:**
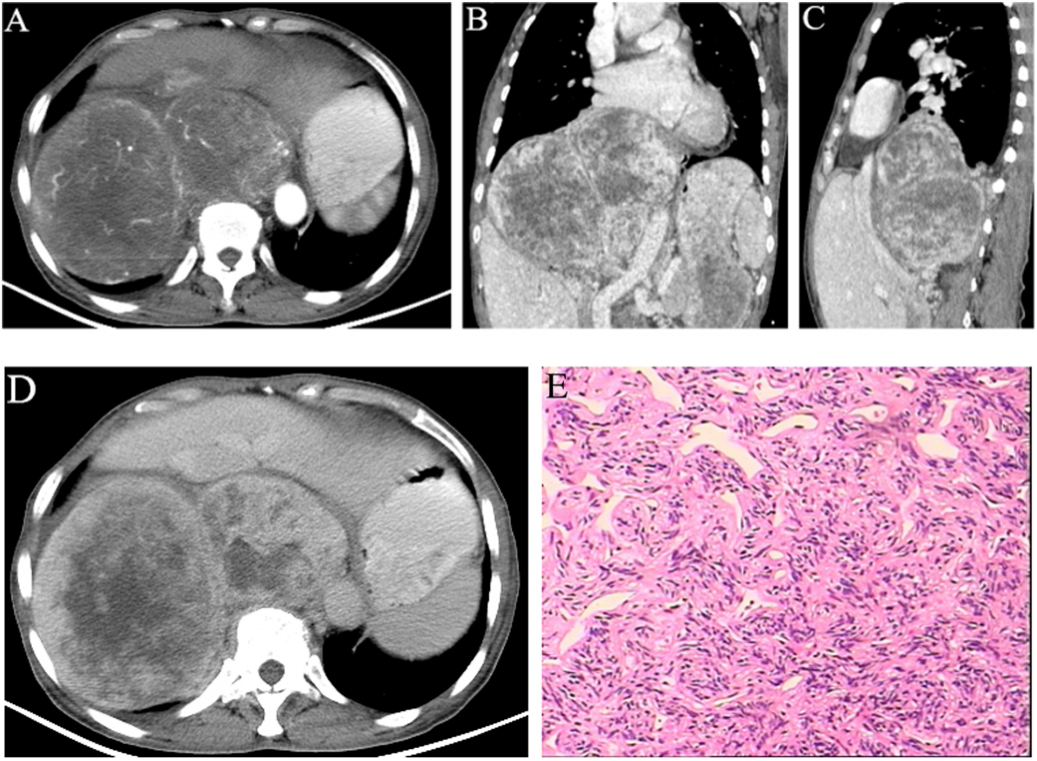
**Right retroperitoneal solitary fibrous tumor in a 61-year-old woman. (A)** Transverse arterial phase shows a huge soft tissue mass fused by multiple nodules of varying sizes, and the solid part is in moderate, uneven enhancement. **(B** **and** **C)** Multi-planar reconstruction in venous phase shows that the solid part of the tumor is in more significant, sustained, uneven enhancement, no enhancement in low-density cystic areas, but envelope enhancement is visible. **(D)** The solid part has sustained enhancement in the delayed phase. **(E)** Tumor cells were fusiform with a bundle-like, spiral arrangement under the microscope, rich sinusoids and interstitial collagen denaturation were observed in the focus areas.

Case two was a 61-year-old female, who developed a huge retroperitoneal placeholder as indicated by examination. The patient had neither chest tightness, shortness of breath, difficulty in breathing, nor abdominal discomfort. Upon physical examination, the abdomen was flat and soft, with no palpable mass. Plain chest and abdominal CT scans showed a huge soft tissue mass shadow fused by multiple nodules of varying sizes on the right retroperitoneum, approximately 164 mm × 150 mm × 116 mm in size. Heterogeneous density and extensive low-density cystic areas were seen, with CT values of 25 to 40 HU, and clear boundaries with the surrounding organs. There was a shift in the heart, and the liver and right kidney were under compression, as well as the aorta and inferior vena cava. Varicose veins in the abdomen were also observed. The solid part of the mass in the arterial phase was moderately and heterogeneously enhanced, with a CT value of about 83 HU, and a plexiform disordered vascular shadow was visible (Figure 2A). The solid part of the scanned mass in the venous and delayed phases was more enhanced in a sustained and heterogeneous manner, with CT values of 99 to 123 HU. No enhancement was observed in low-density cystic areas although a significant enhancement in the envelope was visible. Multi-planar reconstruction displayed the tumor size, location, and relationship with the surrounding tissue more clearly (Figure 2B–D). Due to the high surgical risks as determined by consultation, a retroperitoneal tumor biopsy was performed under CT guidance. The tumor cells were in fusiform shape in a bundle-like, spiral arrangement under the microscope, rich in sinusoids, and interstitial collagen denaturation in the focus area (Figure 2E). Immunohistochemistry showed CD34 (++), bcl-2 (++), CD99 (++), F8 (+); CD117 (-), Desmin (-), S-100 (-), and CK (-). Because of the rich vascular supply and the potential risks of surgery, the mass was treated with embolization. After two subsequent CT examinations, 3 and 6 months later, the tumor was unchanged with regards to size and appearance.

### Case three

**Figure 3:**
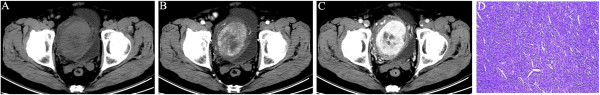
**Retroperitoneal solitary fibrous tumor in a 62-year-old man. (A)** CT scan shows huge retroperitoneal soft tissue mass on the right side of the bladder in uneven density. **(B)** The solid part in arterial phase is in moderate, uneven enhancement, with tumor vessel, and cystic areas of low density are visible inside. **(C)** The solid part in venous phase is in more significant, sustained, uneven enhancement, no enhancement in low-density cystic areas, but envelope enhancement is visible. **(D)** The tumor was composed of rich, dense, fusiform cells under the microscope, with branching vascular channels, collagenous bundles, and coagulation necrosis in focus areas.

Case three was a 66-year-old male, who developed a huge retroperitoneal placeholder in the pelvic cavity, as revealed by examination. The patient had no back pain or hematuria, no abdominal distention or abdominal pain, and no sychnuria, urgency, or dysuria. A plain pelvic CT scan showed a huge retroperitoneal soft tissue mass on the right side of the bladder, approximately 78 mm × 63 mm × 58 mm in size, with a heterogeneous density. Multiple cystic areas of low density were seen, with CT values of 25 to 40 HU, and with unclear boundaries with the neighboring bladder (Figure 3A). The solid part of the mass was moderately and heterogeneously enhanced in the arterial phase, with a CT value of about 95 HU, and a thickened disordered vascular shadow was visible (Figure 3B). The solid part of the scanned mass in the venous phase was further enhanced in a heterogeneous manner, with a CT value of 105 to 136 HU. No enhancement for low-density cystic areas was seen, while a significant envelope enhancement was visible (Figure 3C). The retroperitoneal tumor on the right side of the pelvic cavity was visible in surgery, with adhesions on the left side of the bladder and the right side of the pelvic cavity, and the envelope rich in surface blood vessels. Surgical pathology showed hyperplasia with a bundle-like structure and a storiform arrangement was seen under the microscope. The tumor cells were rich, dense, and in fusiform shape in most areas, but sparse in others. Rich blood vessels were seen in interstitium, with partial interstitial collagen denaturation and coagulation necrosis in a small focus area. Fibrous tissue hyperplasia was seen on the outside of some tumor tissues, with lymphocytic infiltration inside (Figure 3D). Immunohistochemistry showed CD34 (++), CD99 (+), bcl-2 (+), S100 (-), CD117 (-), NSE (-), Desmin (-), and SMA (-).

## Discussion

The onset age of SFTs is 19 to 85 years old, and the age of peak incidence is 40 to 60 years old. SFTs are more common in women. In our study, there were two female patients. Visceral pleura is the most common site of SFT occurrence, but there are more than 1/3 of SFTs occurring outside of the thoracic cavity, including in the head and neck, spinal cord, abdominal and pelvic cavity, respiratory tract and surrounding soft tissue, among others [3–9]. The main clinical manifestations of thoracic SFTs are chest pain, cough, breathing difficulty, low blood sugar, and pulmonary hypertrophic osteoarthropathy. The specific clinical manifestations of extrapleural SFTs depend on the location and size of the tumor. Retroperitoneal SFTs are rare [10, 11], and mainly presents as a painless mass, which can grow very large. The clinical symptoms are mostly caused by the space-occupying tumor mass. In this group, one case visited the doctor due to abdominal pain caused by the huge tumor, and the remaining two cases were asymptomatic and accidentally discovered during physical examination. The gross specimens of typical SFTs were lobulated, tough-quality tumors with clear boundary or with (pseudo) envelope. Pedicles attached to normal tissues were visible in some tumors. A denser adhesion with surrounding tissue could be seen in a small number of tumors. The section was rigid and gray, often with a texturized, spiral-shaped appearance. Myxoid degeneration, hemorrhage, and necrosis were visible, and some may have had calcification. The basic histological changes were as follows: the sizes of tumor cells were relatively consistent, with a small amount of cytoplasm, in short- to long-spindle shape, and homogeneously distributed nuclear chromatin. The typical cell arrangement was patternless. The dense and loose cell zones coexisted and were separated by fibrous interstitium. Rich blood vessels were seen in the tumor, with dilated thick-walled blood vessels in perivascular sclerosis, and a typical hemangiopericytoma-like tumor could be found. Immunohistochemistry showed that 79 to 100% of the tumor cells had positive expression for CD34, which was a highly specific marker of SFTs [12]. Furthermore, positive CD99 and bcl-2 expressions also had a certain diagnostic value, while S-100 expression was negative [13].

CT can be used to identify the focus area and clarify the origin. It is also important in revealing the characteristics of tumor growth to some extent, as well as for efficacy and prognosis evaluation. The retroperitoneal SFT was mostly lobulated and large, in round or oval shape, and grew slowly in an expansive way. The surrounding tissues mostly shifted under compression. In the three cases discussed herein, the maximum diameter was above 160 mm, and a significant extrusion occurred to the surrounding organs. The organs were deformed under compression. Most tumors have intact envelopes, which is an important feature of retroperitoneal SFTs [14]. In all three cases, the enhanced envelope shadow was visible. In plain CT scan, the tumors had a homogeneous or heterogeneous density, which was equal to or slightly higher compared with the muscle density. Two common intratumoral soft tissue components in different densities were closely related to the content of collagen fibers. The cell-sparse zone had an increased density due to the large number of collagen fibers, while the dense zone had reduced density due to the relatively small number of collagen fibers. When cystic degeneration, necrosis, and myxoid degeneration occurred, the tumor density became more heterogeneous [1, 3, 7, 9]. Combining with literature [1, 6, 8], the SFT enhancement patterns are closely related to the densities of tumor blood vessels and tumor cells and the distribution of dense collagen. There were basically three patterns; first, mild enhancement or no enhancement, with a degree of enhancement in each scan phase not exceeding 50% of the plain CT value. The enhancement can be heterogeneous, mostly without necrosis. Second, moderate enhancement, with an enhancement degree of 50 to 100%, and the enhancement can be homogeneous or heterogeneous, with necrosis rarely seen. Third, significant enhancement, with an enhancement degree greater than 100%. This enhancement pattern is similar to that of hemangiopericytoma, with heterogeneous enhancement and relatively common necrosis. Dynamic enhancement is mostly sustained enhancement or progressively delayed enhancement with a long duration. The blood vessels visible within the tumor are visible in the arterial phase. In the three cases, the scans showed heterogeneous density, with no calcification. The solid part of the tumor was significantly and heterogeneously enhanced, with an enhancement degree exceeding 100%, and the enhancement was sustained. The enhancement features of retroperitoneal SFTs in our group were in agreement with the third type. They were similar to the enhancement features of abdominal and retroperitoneal SFTs reported in the literature [7, 9, 14]. Such an enhancement pattern is related to a variety of patterns of histological arrangement. The cell dense zone and the hemangiopericytoma-like area have a significant enhancement, while the cell sparse zone and the collagen fiber hyaline degeneration zone have relatively weak enhancement. Areas with different enhancement degrees are mixed. Myxoid degeneration and the loose arrangement of the cells can cause the expansion of extracellular space. The contrast agent in the extracellular space has progressive accumulation but slower expurgation, which leads to sustained enhancement. A plexiform, disordered, thickened, and increased vascular shadow was seen in the arterial phase, which reflects the presence of a hemangiopericytoma-like tumor pathologically.

MRI can accurately show the histological features of retroperitoneal SFTs, which has great value for tumor diagnosis. However, the three cases discussed herein did not undergo this procedure. According to the literature [1, 4, 5, 7, 9, 10], T1WI generally had equal or slightly lower signals compared with muscle, while T2WI signals were in large variations and could be represented by a variety of signals. Equal or slightly high signals, and internally scattered flake-like or nodular low signals were the typical MRI manifestations for most SFTs. T2WI high signals reflected the myxoid degeneration area in the tumor, slightly high signals reflected the cell dense zone in the tumor, and low signals reflected the dense collagen fibers. Due to inconsistencies in SFT vessels, intra-tumor cells, and dense collagen in terms of density, the enhanced tumors can show a variety of enhancement patterns, from mild to significant enhancement. Most abdominal SFTs show moderate and above enhancement. Irregular necrosis areas are visible in huge tumors.

Gastrointestinal stromal tumors, neurogenic tumors, and soft tissue sarcoma, among others, need to be distinguished from retroperitoneal SFTs.

## Conclusions

In short, retroperitoneal SFTs are rare, with no typical clinical manifestations, and therefore it is often difficult to make a correct preoperative diagnosis. CT imaging of retroperitoneal SFTs usually shows the following characteristics: large tumors with clear boundaries, intratumoral solid components in two different densities, cystic degeneration, necrosis, and myxoid degeneration, significant enhancement and a visibly enhanced envelope. These features can be used as the criteria for differential diagnosis for tumors originating from the retroperitoneum. The confirmed diagnosis still depends on histological and immunohistochemical examinations.

## Consent

Written informed consent was obtained from the patient for the publication of this report and any accompanying images.

## Abbreviations

SFT: solitary fibrous tumor
CT: computed tomography.
